# Anti-Inflammatory Activity of A Polyphenolic Extract from *Arabidopsis thaliana* in In Vitro and In Vivo Models of Alzheimer’s Disease

**DOI:** 10.3390/ijms20030708

**Published:** 2019-02-07

**Authors:** Roberto Mattioli, Antonio Francioso, Maria d’Erme, Maurizio Trovato, Patrizia Mancini, Lucia Piacentini, Assunta Maria Casale, Ludger Wessjohann, Roberta Gazzino, Paolo Costantino, Luciana Mosca

**Affiliations:** 1 Department of Biology and Biotechnology “Charles Darwin”, Sapienza University of Rome, 00185 Roma, Italy; maurizio.trovato@uniroma1.it (M.T.); lucia.piacentini@uniroma1.it (L.P.); assuntamaria.casale@uniroma1.it (A.M.C.); robertagazzino@gmail.com (R.G.); paolo.costantino@uniroma1.it (P.C.); 2 Department of Biochemical Sciences, Sapienza University of Rome, 00185 Roma, Italy; maria.derme@uniroma1.it (M.d.); luciana.mosca@uniroma1.it (L.M.); 3 Department of Bioorganic Chemistry, Leibniz Institute of Plant Biochemistry, 06120 Halle (Saale), Germany; Ludger.Wessjohann@ipb-halle.de; 4 Department of Experimental Medicine, Sapienza University of Rome, 00185 Roma, Italy; patrizia.mancini@uniroma1.it

**Keywords:** *Arabidopsis thaliana*, amyloid beta, polyphenols, Alzheimer’s disease, BV2 cells, interleukins, Nrf2, NF-κB

## Abstract

Alzheimer’s disease (AD) is the most common neurodegenerative disorder and the primary form of dementia in the elderly. One of the main features of AD is the increase in amyloid-beta (Aβ) peptide production and aggregation, leading to oxidative stress, neuroinflammation and neurodegeneration. Polyphenols are well known for their antioxidant, anti-inflammatory and neuroprotective effects and have been proposed as possible therapeutic agents against AD. Here, we investigated the effects of a polyphenolic extract of *Arabidopsis thaliana* (a plant belonging to the Brassicaceae family) on inflammatory response induced by Aβ. BV2 murine microglia cells treated with both Aβ_25–35_ peptide and extract showed a lower pro-inflammatory (*IL-6*, *IL-1β*, *TNF-α*) and a higher anti-inflammatory (*IL-4*, *IL-10*, *IL-13*) cytokine production compared to cells treated with Aβ only. The activation of the Nrf2-antioxidant response element signaling pathway in treated cells resulted in the upregulation of heme oxygenase-1 mRNA and in an increase of NAD(P)H:quinone oxidoreductase 1 activity. To establish whether the extract is also effective against Aβ-induced neurotoxicity in vivo, we evaluated its effect on the impaired climbing ability of AD Drosophila flies expressing human Aβ_1–42_. Arabidopsis extract significantly restored the locomotor activity of these flies, thus confirming its neuroprotective effects also in vivo. These results point to a protective effect of the Arabidopsis extract in AD, and prompt its use as a model in studying the impact of complex mixtures derived from plant-based food on neurodegenerative diseases.

## 1. Introduction

Natural polyphenols, a vast group of compounds present in plant-based food, confers protection against cancer, cardiovascular, metabolic and neurodegenerative diseases [1,2,3,4].

The interest in polyphenols as possible therapeutic agents in neurodegenerative diseases stems from the well-documented evidence of their antioxidant effects both in vitro and in vivo, as largely studied in Alzheimer’s disease (AD) [5]. The complex neuropathology of AD is primarily characterized by increased production of amyloid-β (Aβ) oligomeric species and extracellular deposition of fibrillary Aβ peptides in the form of plaques, whose accumulation is a major cause of inflammation, oxidative stress and mitochondrial dysfunction [6,7]. Among the various neuroprotective strategies against Aβ-mediated reactive oxygen species (ROS) damages and inflammation proposed in AD, polyphenols represent an attractive therapeutic option [8].

Indeed, polyphenols may counteract neurodegenerative diseases with different mechanisms at various levels. They exert antioxidant activity by inducing the expression of antioxidant enzymes, as well as inhibiting the expression of pro-oxidant enzymes [9,10,11]. By modulating intracellular signaling cascades and gene expression, and by interacting with mitochondria, they induce morphological changes that have a direct positive influence on memory acquisition, consolidation and storage. Moreover, polyphenols exert a remarkable anti-inflammatory activity by suppressing the activation of microglia, and by affecting inflammatory redox signaling pathways via modulation of pro-inflammatory gene expression, mainly acting through nuclear factor-kappaB (NF-κB) and mitogen-activated protein kinase signaling [9,12,13]. Furthermore, polyphenols may modulate Aβ production and aggregation by stimulating α-secretase and by inhibiting β- and γ-secretase, and by competitively interacting with aromatic residues preventing the π–π interaction, thus blocking the amyloid self-assembly process [14,15]. Some phenolics, such as quercetin and myricetin, are able to inhibit the β-secretase enzyme interfering with the Aβ generation cascade, whereas other phenolics, such as epigallocatechin gallate, curcumin, *trans*-resveratrol, or oleuropein, are able to inhibit amyloid aggregation and fibrillogenesis [16]. A polyphenol rich extract of Hypericum was suggested to positively affect Aβ clearance by activation of ABC transporters in the blood-brain-barrier [17,18,19].

Polyphenols have the capacity to influence gene expression by modulating epigenetic mechanisms that have been associated with AD [20]. For instance, resveratrol is an indirect activator of SIRT-1, an NAD^+^-dependent deacetylase found to be reduced in the cortex of patients with AD, whereas curcumin was shown to inhibit both histone acetyl transferase and DNA methyl transferase-1 [21].

A large body of literature suggests that inflammation is largely mediated by activated microglia in neurodegenerative diseases [22,23]. It has been hypothesized that dietary interventions through the consumption of foods rich in polyphenols and other antioxidants and anti-inflammatory agents may prevent the onset of age-related neurodegenerative diseases [24]. However, in spite of their efficacy, demonstrated by many in vitro studies, single isolated polyphenols have displayed a limited effect in vivo and in clinical trials, mainly due to low bioavailability and scarce solubility [25,26]. Conversely, mixed compounds in food sources have unequivocal beneficial effects, and this might be attributed to additive and even synergistic effects among different polyphenols and other food components, such as lipids, sterols, vitamins and minerals. Indeed, there is a growing interest in the importance of assessing the interactions among the various phytochemicals in food, with regard to their effects on the pathophysiology of the human body. However, relatively few studies have yet taken into account the potential protective effects of complex matrices of bioactive molecules contained in foods (for a review see [27]).

Here, we investigated the effects of a polyphenolic extract from the model plant *Arabidopsis thaliana* on the inflammatory response in BV2 cells treated with Aβ_25–35_ peptide and in an animal model of AD, i.e., *Drosophila melanogaster* flies expressing human Aβ_1–42_. *Arabidopsis thaliana* belongs to the Brassicaceae family and is a model plant widely used in several research fields [28,29]. It grows quickly in a growth chamber with computer-controlled crucial parameters—quality/quantity of light, day and night cycles, humidity and temperature—ensuring uniform growth conditions and composition of the juices. Moreover, Arabidopsis has a fully sequenced and extensively mapped genome and metabolome [30,31], as well as a large collection of mutants and genetic/molecular tools that make metabolic engineering possible, thus representing an appealing system to study the impact of polyphenolic complex matrices on several human disease models. 

## 2. Results

### 2.1. Qualitative and Quantitative Analysis of Polyphenols in Arabidopsis Extract

Several authors reported the presence of flavonoid glycoside derivatives and hydroxycinnamoyl derivatives in Arabidopsis extracts [30,32,33,34]. To establish the qualitative/quantitative composition of our extract, Arabidopsis seedlings were grown for seven days and the raw juice obtained by cold pressure was analyzed by chromatographic or colorimetric methods. As the raw juice may contain also proteins, minerals, nucleic acids and fibers or chlorophyll that may interfere with polyphenolic determination, the raw juice was extracted with ethyl acetate (EtOAc) in order to obtain a selective enrichment in flavonoids. 

**Figure 1:**
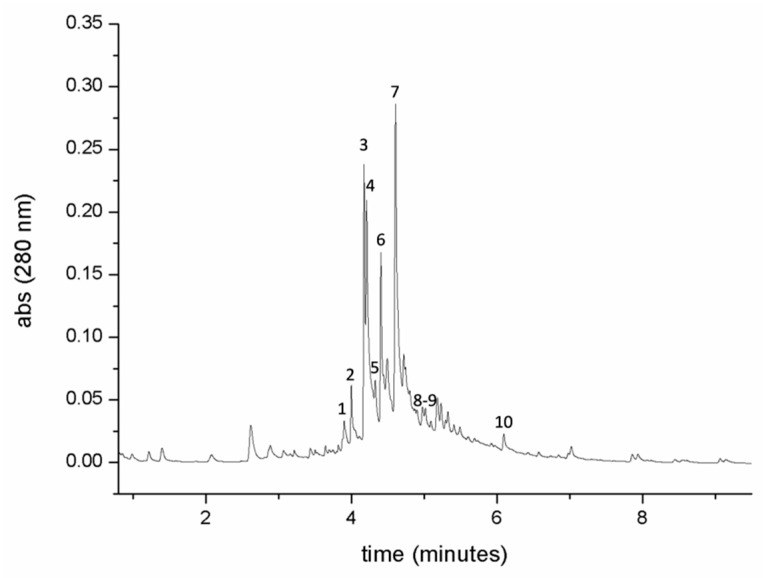
Chromatographic analysis of *Arabidopsis thaliana* seedlings polyphenols extracted with ethyl acetate from raw juice, registered at 280 nm. Peaks numbered 1–10 are reported in Table 1.

Chromatographic analyses of an EtOAc extract of raw juice revealed the presence of polyphenols and flavonoids (Figure 1). Under our chromatographic conditions, the main constituents of the extract were eluted between 4 and 6 min. Main peaks were identified as polyphenols or flavonoids by chromatographic behavior, comparison with analytical standards, UV-visible spectra, molecular mass, molecular formula, and MS/MS fragmentation, and are detailed in Table 1.

Results and experimental data are reported in Table 1. Peak 1 was identified as caffeic acid. Peaks 2–3–4–5 and 6 were identified, respectively, as quercetin-exoside-rhamnoside, kaempferol-3-*O*-glucoside-7-*O*-rhamnoside, quercetin-dirhamnoside, isorhamnetin-exoside-rhamnoside and kaempferol-3,7-*O*-dirhamnoside. Sinapic acid (peak 7) was identified as the major polyphenolic component of EtOAc extract. Peaks 8 and 9 were identified as two isomers of 1,2-di-synapoyl-d-glucose. Luteolin (peak 10) was one of the last eluted compounds, due to its more hydrophobic nature. All our experimental data reported in Table 1 are in agreement with the known and recent reports in the literature [35,36]. For the detailed chemical structures of each compound see Figure S1 supplementary materials.

The total content of polyphenols and anthocyanins was estimated by the Folin-Ciocalteau and pH differential methods, respectively. The analysis revealed that EtOAc selectively extracts the flavonoidic fraction, while anthocyanins and simple phenolics largely remain in the aqueous layer, as demonstrated by chromatographic analyses (not shown). Total polyphenols, expressed as mg GAE/mL, were found to be 0.32 ± 0.02 and 0.10 ± 0.01 for raw juice and EtOAc extract, respectively; whereas total anthocyanins, expressed as μg CGE/mL, were 0.88 ± 0.05 in raw juice and absent in the EtOAc extract. 

In order to verify whether the antioxidant activity was affected by the EtOAc extraction procedure, we performed the 2,2-diphenyl-1-picrylhydrazyl (DPPH) assay on both the raw juice and the extract (see Figure S2 supplementary materials). Data revealed that while in the EtOAc extract the antioxidant activity was linearly related to the amount of extract added, in the raw juice the activity reached a plateau. These results may be justified by the fact that the DPPH assay is performed in ethanol and this solvent may cause the precipitation of proteins and other pigments present in the raw juice that could interfere with spectrophotometric determinations. Based on these observations, all subsequent experiments were performed using the EtOAc extract.

### 2.2. The Arabidopsis EtOAc Extract Affects Pro-Inflammatory and Anti-Inflammatory Cytokines Gene Expression in BV2 Cells

To analyze the inflammatory response, BV2 cells were treated up to 24 h, with 25 μM Aβ_25–35_ aggregated peptide in the presence or absence of the EtOAc extract (20 μL/mL), and the expression of several pro-inflammatory and anti-inflammatory cytokine genes was measured by qRT-PCR. Both the concentration of Aβ_25–35_, as well as the amount of EtOAc extract were chosen based on dose-response curves and on previous data [37,38]. The effect of different amounts of Arabidopsis EtOAc extract on BV2 cellular viability was tested up to 50 μL/mL: No cytotoxic effects were observed up to 20 μL/mL, whereas higher concentrations caused a slight viability decrease (data not shown). Based on these results, we used a concentration of 20 µL/mL of extract for further experiments. 

**Figure 2:**
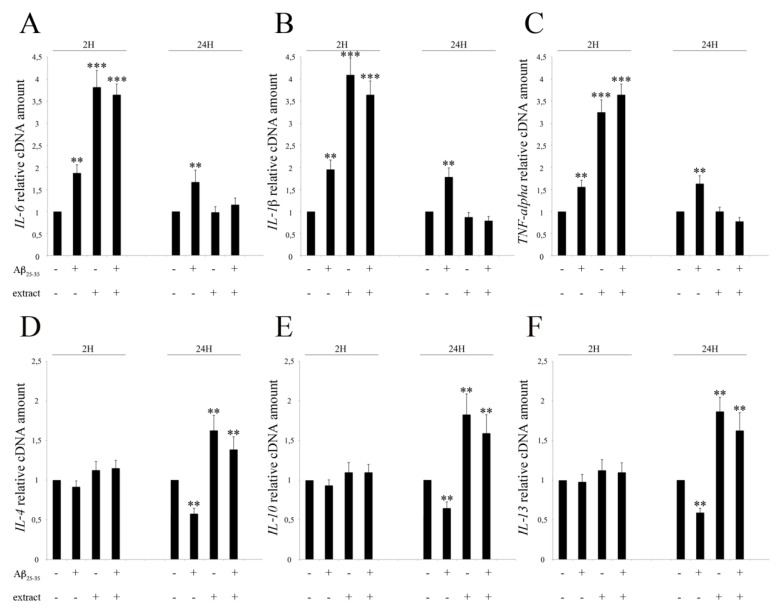
Modulation of pro-inflammatory and anti-inflammatory cytokines in BV2 cells treated with 25 μM Aβ_25–35_ and/or 20 μL/mL of EtOAc extract, evaluated by qRT-PCR at 2 and 24 h. (**A**) *IL-6* expression; (**B**) *IL-1β* expression; (**C**) *TNF-α* expression; (**D**) *IL-4* expression; (**E**) *IL-10* expression; (**F**) *IL-13* expression. Data are shown as mean ± SEM (*n* = 3). ** *p* < 0.01 vs. Ctrl; *** *p* < 0.001 vs. Ctrl.

Figure 2 reports the expression of cytokine genes (*IL-6*, *IL-1β*, *TNF-α*, *IL-4*, *IL-10*, *IL-13*) in control and treated samples at 2 and 24 h. As shown in Figure 2A–C, at 2 hours, *IL-6*, *IL-1β* and *TNF-α* showed overexpression in samples treated with 25 μM Aβ_25–35_ peptide (about 1.5–2 fold), and an even higher expression in samples treated with the EtOAc extract or with both Aβ_25–35_ peptide and EtOAc extract compared to control (about 3–4 fold). The statistical analyses indicate that the overexpression was highly significant, with *p* < 0.01 vs. control for samples treated with 25 μM Aβ_25–35_ peptide and with *p* < 0.001 vs. control for samples treated with EtOAc extract or with both Aβ_25–35_ peptide and EtOAc extract.

After 24 h, pro-inflammatory cytokines gene expression remained steadily high in BV2 cells treated with Aβ_25–35_. Conversely, in cells treated with the EtOAc extract, gene expression reverted to the level of untreated controls, both in the presence and in the absence of Aβ_25–35_. In particular, no significant differences could be observed between controls and samples treated with either the EtOAc extract alone or in the presence of Aβ_25–35_, whereas the treatment with Aβ_25–35_ alone maintained the expression values of all cytokine genes at around 1.5–2 times higher than control, with *p* < 0.01 vs. control. The lower pro-inflammatory cytokine gene expression observed after 24 h in samples treated with either the EtOAc extract or the extract and Aβ_25–35_, was already appreciable after six hours (data not shown).

We also analyzed the anti-inflammatory response, testing the expression of the anti-inflammatory cytokine genes *IL-4*, *IL-10* and *IL-13* (Figure 2C,D,F). After 2 h, no significant differences between control and samples treated with 25 μM Aβ_25–35_ could be evidenced, although there was a slight decrease of gene expression. After 24 h, the treatment with Aβ_25–35_ peptide gave rise to a significant decrease of gene expression in all genes considered, with values of about 0.6 compared to control (*p* < 0.01). In samples treated with the EtOAc extract, either alone or in the presence of Aβ_25–35_, the expression of anti-inflammatory cytokine genes increased by about 1.5–2 fold compared to controls at 24 h, while after 2 h only a non statistically significant increase was evidenced, indicating a positive trend.

### 2.3. The Arabidopsis EtOAc Extract Affects Nrf2 and p65 Nuclear Translocation in BV2 Cells

**Figure 3:**
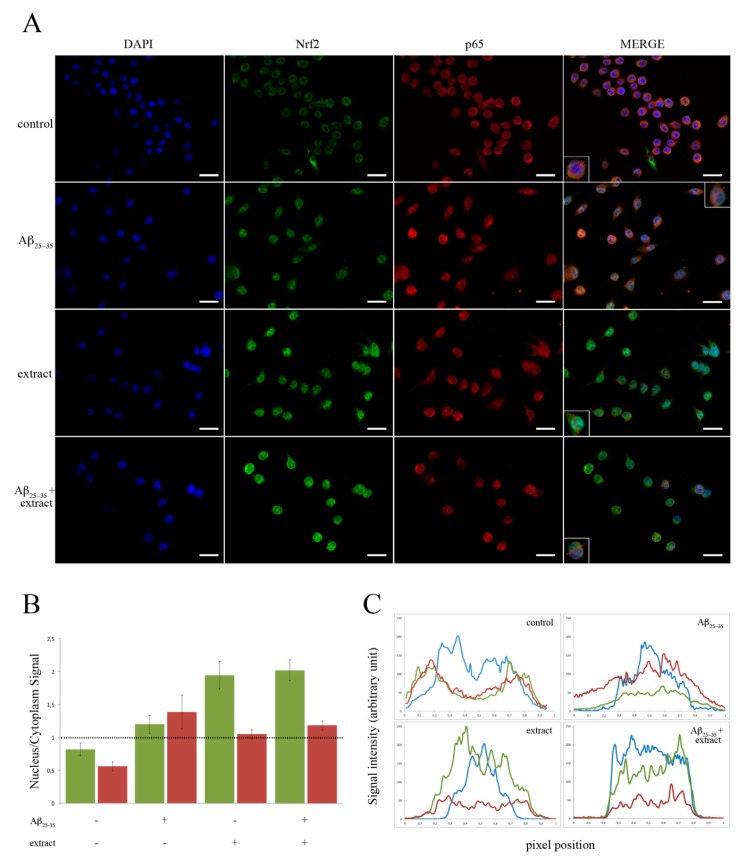
Nuclear translocation of Nrf2 and NF-κB. BV2 cells were incubated with 25 μM Aβ_25–35_ in the presence or in the absence of 20 μL/mL of EtOAc extract. (**A**) Representative images of Nrf2 and NF-κB nuclear translocation verified by immunofluorescence microscopy by utilizing anti-Nrf2 (green) and anti-p65 (red) antibodies and DAPI staining (blue) (scale bar = 50 μm). (**B**) Densitometric analysis of the nuclear/cytoplasm signal ratio as calculated by analyzing immunofluorescence images by ImageJ software (*n* = 50). Green bar = Nrf2; Red bar = p65. (**C**) Analysis by fluorescence staining intensity vs. pixel position of a virtually cut representative cell (The virtual cut is shown by a red line in the insets in panel (**A**)). Blue line = DAPI; Red line = p65; Green line = Nrf2.

To elucidate the upstream signaling pathway involved in EtOAc extract-induced upregulation of anti-inflammatory and downregulation of pro-inflammatory cytokine genes, we focused on the activation of Nrf2 (nuclear factor E2-related factor 2) and NF-κB (nuclear factor kappa-light-chain-enhancer of activated B cells). We investigated the Nrf2 and p65 (a subunit of NF-κB complex) nuclear translocation by immunofluorescence upon treatment of BV2 cells with the Aβ_25–35_ peptide, with the EtOAc extract, or both compared to untreated cells (Figure 3A). After 2 h of incubation, most of the Nrf2 and p65 signal in control samples was localized outside the nucleus, with a nucleus/cytoplasm (*n*/*c*) signal ratio of 0.83 ± 0.09 for Nrf2 and 0.57 ± 0.07 for p65 (Figure 3B). When BV2 cells were treated with 25 μM Aβ_25–35_ peptide, a small fraction of Nrf2 translocated into the nucleus and the n/c signal ratio became 1.18 ± 0.13. Conversely, the *n*/*c* signal ratio of p65 raised from 0.57 to 1.37 ± 0.25, indicating a major change in p65 localization and the activation of NF-κB-mediated pathways. In cells treated with the EtOAc extract alone or in combination with the Aβ_25–35_ peptide, the nuclear translocation of these proteins was more pronounced: The n/c ratio of Nrf2 raised from 0.83 in the control, to 1.94 ± 0.20 in the former condition and to 2.02 ± 0.15 in the latter; as of p65, the n/c raised from 0.57 in control samples to 1.05 ± 0.07 and to 1.20 ± 0.07 in samples treated with the EtOAc extract alone or in combination with the Aβ_25–35_ peptide, respectively.

Analysis by fluorescence staining intensity vs. pixel position showed similar results (Figure 3C). In particular, in control samples the Nrf2 and p65 signal intensity (expressed in arbitrary units—au) was very low in the nucleus (0.2–0.8 pixel range), but was higher than control in samples treated with Aβ_25–35_, reaching maximum values of 65 au for Nrf2 and 155 au for p65 in the nucleus. Conversely, in cells treated with the EtOAc extract alone or in combination with Aβ_25–35_, the nuclear translocation of Nrf2 reached maximum values of 250 and 230 au, respectively, whereas p65, was less evident, with maximum values of 50 and 90 au, respectively.

All in all, these data suggest the activation of the NF-κB pro-inflammatory pathway in samples treated with Aβ_25–35_ and the activation of Nrf2 anti-inflammatory pathway in samples treated with the EtOAc extract alone or in combination with Aβ_25–35_.

### 2.4. The Arabidopsis EtOAc Extract Affects Cellular Antioxidant Defense Capacity and Counteracts Aβ_25–35_ Induced Cytotoxicity

**Figure 4:**
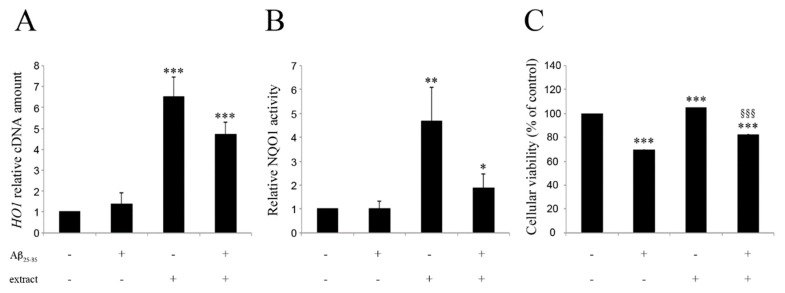
Modulation of HO-1 expression, NQO1 activity and cellular viability of BV2 cells treated with 20 μM Aβ_25–35_ and/or 20 μL/mL of EtOAc extract. (**A**) *HO-1* expression at 2 h. (**B**) Measurement of NQO1 activity at 24 h. (**C**) Cellular viability at 24 h (% of control). Data are shown as mean ± SEM (*n* = 3). * *p* < 0.05 vs. Ctrl; ** *p* < 0.01 vs. Ctrl; *** *p* < 0.001 vs. Ctrl; §§§ *p* < 0.05 vs. Aβ_25–35_.

It is well known that the activation of the Nrf2 pathway mediates the activation of a set of genes that modulate the antioxidant response of the cell. Among these genes two of the most readily responsive are those encoding the heme oxygenase (HO-1) and the DT-diaphorase (NAD(P)H:quinone oxidoreductase 1, NQO1). To verify whether the nuclear translocation of Nrf2 activates the antioxidant cellular response, we determined the mRNA levels of *HO-1* by qRT-PCR. Moreover, we tested the activity of the enzyme NQO1 by means of a specific spectrophotometric assay. As shown in Figure 4A, in BV2 cells treated with 25 μM Aβ_25–35_ for 2 hours *HO-1* gene expression showed little change, compared to untreated controls (1.4-fold increase, not statistically significant), while it showed a substantial increase in BV2 cells treated with the EtOAc extract alone or in combination with Aβ_25–35_ (6.5- and 4.7-fold, respectively; *HO-1*, *p* < 0.001 vs. control).

Measurement of NQO1 activity levels in BV2 cells did not show any effect of Aβ_25–35_ treatment (Figure 4B). Conversely, treatment with the EtOAc extract, either alone or in the presence of Aβ_25–35_, caused a significant increase of the NQO1 activity compared to controls (3.5 and 2 fold, respectively), pointing to an antioxidant effect of the extract.

In order to confirm a cytoprotective effect of the extract, we analyzed the viability of BV2 cells by MTT assay, in the presence of Aβ_25–35_, of EtOAc extract and of both. As shown in Figure 4C, 25 μM Aβ_25–35_ treatment significantly decreased BV2 cells viability as compared to untreated cells (65% at 24 h, *p* < 0.001 vs. control), while the treatment with the EtOAc extract alone did not affect cell viability (106% at 24 h, *p* < 0.001 vs. control). Co-treatment with EtOAc extract and Aβ_25–35_ significantly reduced Aβ_25–35_-induced cytotoxicity (80% at 24 h, *p* < 0.001 vs. Aβ_25–35_), pointing to a protective effect of the extract.

### 2.5. The Arabidopsis EtOAC Extract Alleviates the Locomotor Dysfunction Induced by Aβ_1–42_ in Drosophila melanogaster

**Figure 5:**
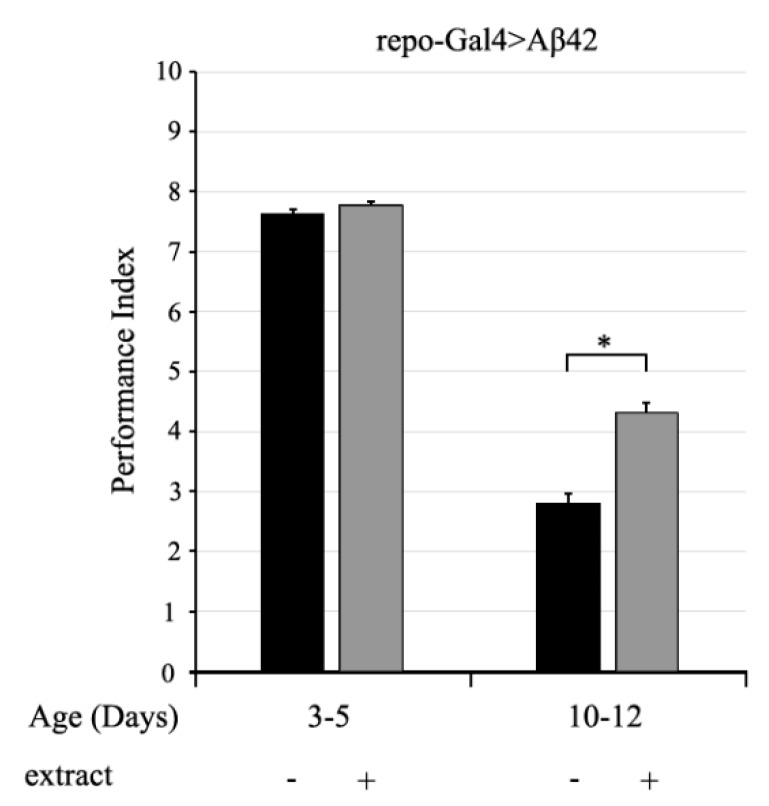
Alzheimer’s disease (AD) flies expressing Aβ_1–42_ in glial cells using the repo-Gal4 driver were allowed to feed on the diet supplemented with EtOAc extract (40 μL/mL in standard sugar-yeast medium) for the entire developmental period and then assayed for climbing activity at 3–5 and 10–12 days. Climbing abilities are presented as the average performance index (PI) ± SEM. * *p* < 0.05.

In order to study the neuroprotective and therapeutic potential of the complex mixtures of polyphenols in vivo, we evaluated the climbing ability of transgenic AD flies expressing human Aβ_1–42_ fed on standard medium supplemented with polyphenolic extract (Figure 5). In our experiments, the glial-specific expression of the human pathogenic construct (Aβ_1–42_) under the control of UAS (Upstream Activator Sequence) elements was achieved by using the pan-glial driver repo-Gal4 [39]. AD flies expressing Aβ_1–42_ were allowed to feed on the diet supplemented with the EtOAc extract (40 μL/mL in standard sugar-yeast medium) for the entire developmental period and then assayed for climbing activity at 3–5 and 10–12 days post-eclosion. The results obtained showed that the EtOAc extract significantly ameliorates impaired climbing ability of Aβ_1–42_ flies when compared to untreated control flies, confirming that the Arabidopsis polyphenolic extract exerts a neuroprotective effect also in vivo.

## 3. Discussion

The inflammatory response of the brain is mediated by its resident microglia which becomes “activated” when a noxious stimulus hits the brain [22]. Neuroinflammation is initially a protective defense mechanism against injuries or infective agents. In AD brains, Aβ induces increased production of pro-inflammatory cytokines, such as IL-1β, TNF-α and IL-6, which have been demonstrated to cause neuronal toxicity and death [40]. The production of pro-inflammatory cytokines, while having some beneficial effect by ameliorating amyloid deposition, can induce an “autotoxic loop” and enhance tau pathology and neurodegeneration [22]. Based on these observations, it has been proposed that anti-inflammatory drugs may have a therapeutic role in AD. A large body of literature supports the efficacy of NSAIDs in counteracting Aβ toxicity and production, however, clinical trials in AD patients failed to evidence any beneficial effect [23]. 

The use of polyphenols has been proposed as an alternative valid strategy to dampen inflammatory processes in early AD, and therefore to reduce AD severity. These natural compounds are abundantly present in some foods and, more importantly, food matrices contain mixtures of different polyphenols and flavonoids, which can have additive or even cooperative effects.

Recently, we demonstrated that juices from *Brassica oleracea* sprouts, containing a complex mixture of polyphenols, are able to protect neuronal cells in culture from the deleterious effects of Aβ_25–35_, through antioxidant mechanisms mediated by the activation of the nuclear factor Nrf2 [41]. The juices derived from edible plants, such as *Brassica oleracea*, hardly represent the ideal tool to understand how a bioactive compound acts, or how several bioactive molecules interact, in affecting a biological process. Indeed, the domestication of plants resulted in changes to several traits and reduced the genetic variability for several other: In particular healthy bitter components, like many flavonoids or mustard oil components, have been strongly reduced or even eliminated by breeding practices. Most wild plant species, including *Arabidopsis thaliana*, show a solid intraspecific natural variation for several properties involved in adaptation to different environmental growth conditions (for a review see [42]). Each of the about 7000 wild type Arabidopsis accessions collected and available in the stock center [43] may be characterized by a different polyphenolic profile as suggested by Routaboul et al. [44]. Moreover, *Arabidopsis thaliana* can be grown in growth chambers under strictly controlled environmental conditions (light, temperature, humidity etc.). As growth conditions do affect the production of secondary metabolites, varying Arabidopsis growth parameters allow to reproducibly modify the polyphenolic content of this plant. Furthermore, the genome of Arabidopsis is completely sequenced and extensively mapped, and a large collection of mutants, as well as a vast arsenal of genetic/molecular tools are available, making metabolic engineering feasible.

Here, we investigated the efficacy of complex polyphenolic mixtures as anti-inflammatory agents in a cellular model of inflammation in AD, i.e., BV2 murine microglial cells stimulated with Aβ_25–35_ in the presence or absence of a polyphenolic extract from *Arabidopsis thaliana* seedlings.

Polyphenol-containing juice was obtained by cold pressure of seedlings grown for seven days. The choice of this starting material was based on the observation that seedlings usually contain much higher amounts of bioactive and protective compounds per gram of fresh weight than adult plants. Cold pressure was performed to limit auto-oxidation of the phenolic compounds, due to high temperature. Before proceeding to the qualitative analysis of the Arabidopsis extract by UPLC-MS-MS and test them on the cellular and animal models of AD, we enriched the extract in the flavonoidic fraction by extraction with EtOAc. This procedure also allowed us to eliminate proteins, minerals, nucleic acids and fibers or chlorophyll which could affect the interpretation of results. The analysis of phenolics, performed in both raw juices and purified EtOAc extract, revealed the presence of caffeic acid, kaempferol and quercetin derivatives, along with sinapic acid and its derivatives. These results indicate that the Arabidopsis seedling juice has a polyphenolic content similar to that of seeds or of several tissues of adult plants [30,36].

By using this EtOAc extract, we assessed the gene expression profile of pro-inflammatory and anti-inflammatory cytokines following Aβ treatment in the presence or absence of the EtOAc extract in the short and in the long term. The expression of genes encoding anti-inflammatory cytokines (*IL-4*, *IL-10*, *IL-13*) was downregulated following Aβ treatment, whereas it was upregulated when the phenolic extract was used -either alone or in the presence of Aβ_25–35_, pointing to an action of these compounds against the effects of Aβ_25–35_. Conversely, the expression of pro-inflammatory cytokines (*IL-6*, *TNF-α*, *IL-1β*) increased when the cells were treated with Aβ_25–35_, whereas the effect of the EtOAc extract was less linear. In fact, in the short term the extract seemed to enhance the expression of the cytokines, while in the long term it restored the expression to control levels. Apparently, in the short term the extract is able to activate an inflammatory response which is already reverted at six hours, leading to a suppression of the inflammatory pathway in the long term which contrasts amyloid action. These data may be best interpreted in light of recent evidence which indicates that the production of pro-inflammatory cytokines plays a pivotal role in neurodegeneration, but also in neuroprotection [45]. Indeed, microglia are able to exert either neuroprotective or neurotoxic effects depending on its activation state. Notably, microglia could assume a “classically activated” state, termed M1 (a definition borrowed from the activated macrophage states), which is induced by the presence of TNF-α or INFγ produced by T-cells or natural killer cells, and displays a high defense profile against pathogens by acidifying the phagosome and releasing ROS/RNS. Conversely, the activation state M2, which results from stimulation of glia by IL-4 and -13, or by IL-10, gives rise to wound healing or anti-inflammatory type microglia. The delicate balance between these two activation states drives the evolution of the neurodegenerative disease. The M1 state is not dangerous *per se*, but could rather play a defense role in the first phases of the disease. However, if the activation of the M1 state persists and is not replaced by M2 state microglia, the prolonged inflammatory stimulus results in a deleterious effect and tissue damage [46]. 

The production of cytokines is under the control of a signal transduction circuit involving the Nrf2 and NF-κB nuclear factors. Nrf2 is a transcription factor that regulates the expression of antioxidant genes that protect against oxidative damage, injury and inflammation by stimulating the anti-inflammatory cytokine genes and antioxidant or phase II detoxification enzymes. This transcription factor is activated by various compounds present in Brassicaceae, such as polyphenols and glucosinolates [47]. Conversely, NF-κB, a protein complex formed by p65 and p50 subunits, is involved in pro-inflammatory processes and cell death. The stimulation of BV2 cells by Aβ_25–35_, either alone or in combination with the EtOAc extract, causes nuclear translocation of both Nrf2 and NF-κB p65 subunit within two hours of treatment. In particular, both factors are barely detected at the nuclear level in control cells, whereas under Aβ_25–35_ treatment there is a massive translocation of p65 and a slight translocation of Nrf2. Under polyphenol treatment or co-treatment, Nrf2 translocation is very evident while p65 is still appreciable. The simultaneous presence of Nrf2 and NF-κB p65 in the nucleus when the cells are stimulated with polyphenols, either alone or in the presence of Aβ_25–35_, could be the driving force that stimulates the large expression of the pro-inflammatory cytokines within the short term. According to [48,49], the promoters of pro-inflammatory cytokine genes contain responsive elements for both p65 and Nrf2. However, in the long term the equilibrium is in favor of an anti-inflammatory profile. This shift could be due to an indirect inhibitory effect of Nrf2 on pro-inflammatory cytokines production exerted through the binding to the promoter(s) of alternative genes or to the action of other transcription factors [48].

In short, while in Aβ_25–35_-treated cells the NF-κB pro-inflammatory pathway is prevalent, the Nrf2 anti-inflammatory pathway is predominant in cells co-treated with Aβ_25–35_ and the EtOAc extract or treated with this latter alone. This view is supported by viability data and by the modulation of the expression of genes directly under the control of the Nrf2 pathway, i.e. *HO-1* and *NQO1*. Indeed, cell viability is restored to levels similar to control, and the expression of *HO-1* and the activity of NQO1 are positively regulated, when the cells are co-treated or treated with the EtOAc extract, and the expression of *HO-1* and the activity of NQO1 is positively regulated, whereas both enzymes remain unaltered when the cells are treated with Aβ_25–35_ alone.

In order to confirm the efficacy of the extract in vivo, we performed an experiment in an animal model of AD, i.e. *Drosophila melanogaster* flies expressing the human Aβ_1–42_ peptide at the glial level. Drosophila offers a powerful in vivo model to study the Aβ-induced toxicity, because transgenic expression of the human pathogenic fragment of APP (Aβ_1–42_) recapitulates the majority of pathological hallmarks of AD, including neurotoxicity, nuclear inclusion formation, age-dependent neurodegeneration, locomotor dysfunction and early death [50,51,52,53,54]. The Drosophila AD model has been studied to evaluate the protective effects of selected polyphenols as reported in the comprehensive review by Del Rio and Pardo [55]. For instance, beneficial effects of quercetin are mediated by a protein associated with the cell cycle pathway in the Drosophila AD model driving Arctic Aβ_1–42_ expression in the brain [56], whereas Drosophila transgenes fed with curcumin showed a lower Aβ neurotoxicity, due to an acceleration of amyloid fibril conversion [57]. Moreover, AD flies treated with kaempferol showed a delay of climbing ability loss, a rescue of memory and reduced oxidative stress [58]. In our model of transgenic AD flies expressing human Aβ_1–42_, feeding on standard medium supplemented with polyphenolic extract resulted in a significant improvement of the impaired climbing ability of AD flies, confirming the neuroprotective effect of the EtOAc extract, also in vivo. These observations also corroborate the notion that in this extract different polyphenols act synergistically and better recapitulate the food content.

## 4. Materials and Methods 

### 4.1. Chemicals

Unless otherwise stated, reagents were purchased from Sigma Aldrich (St. Louis, MO, USA). Aβ_25–35_ was synthesized by conventional solid phase chemistry [59]. Other reagents were: Trizol reagent from Invitrogen (Carlsberg, CA, USA); QuantiTect Reverse Transcription kit from QIAGEN (Hilden, Germany); primary antibody rabbit monoclonal anti-Nrf2 and secondary antibody goat polyclonal anti-rabbit IgG (Alexa Fluor^®^ 488) were from Abcam (Cambridge, UK); mouse monoclonal anti-NFκB p65 subunit was from Cell Signaling Technology (Milan, Italy); and anti-mouse IgG (Texas Red) Jackson Immunoresearch Laboratories (West Grove, PA, USA). Tissue culture medium and serum were from Gibco BRL (Life Technologies Inc., Grand Island, NY, USA)

### 4.2. Plant Growth and Juice Preparation

Wild-type early flowering *Arabidopsis thaliana* from Columbia-0 (Col-0) ecotype was used in this work. All plants were grown in a growth chamber at 24/21 °C with a light intensity of 300 μE·m^−2^·s^−1^ under 16 h light and 8 h dark per day. Seeds were surface sterilized with a 2.5% aqueous solution of INOV’chlore (Inov Chem) for 10 min and then rinsed four times with sterile water. After five days of cold treatment, *Arabidopsis thaliana* seeds were plated on one-half MS (Murashige and Skoog medium, Duchefa) and grown in a climate chamber for seven days. The seedlings were collected and cold-pressed for the production of raw juice. The juice obtained was centrifuged at 12,000× *g* for 10 min at 4 °C. The supernatant was immediately frozen in liquid nitrogen and stored at −80 °C until use. 2 mL aliquots were extracted four times with an equal volume of EtOAc. Upper organic phases derived from extractions where combined and divided in 20 aliquots (each corresponding to 100 μL of original extract) in 2 mL propylene microtubes. Vials were then dried under vacuum and each aliquot resuspended in complete culture medium to obtain a final concentration corresponding to 20 μL of original extract per mL of culture medium. Before treating the cells, the diluted extract was filtered on a 0.2 μm sterile cellulose acetate membrane.

### 4.3. Determination of Total Phenols and Anthocyanins

Total reductants (mostly phenols) were determined by the Folin-Ciocalteu assay as described by Singleton et al. [60]. Briefly, the reaction solution was prepared by mixing 10 μL of blank, standard or sample with 790 μL of distilled water. After addition of 50 μL Folin-Ciocalteu reagent the reaction mixture was incubated for 3 min at room temperature (RT) and then 150 μL of a 20% (*w*/*v*) Na_2_CO_3_ aqueous solution were added. After 2 h of incubation, the absorbance at 760 nm was measured on a Hitachi U2000 spectrophotometer (Hitachi, Tokyo, Japan). The results were expressed as mg of gallic acid equivalents (GAE) per mL of juice.

Total anthocyanins quantification was performed by the pH-differential method as described by Giusti and Wrolstad [61]. The juice was diluted in a pH 1.0 solution (0.1 M HCl, 25 mM KCl) and in a pH 4.5 solution (0.4 M CH_3_COONa). The absorbance of the mixtures was then measured at 535 and 700 nm against distilled water. The value (Abs535 − Abs700)pH 1.0 − (Abs535 − Abs700)pH 4.5 corresponds to the absorbance, due to the anthocyanins. Calculation of the anthocyanin concentration was based on a cyanidin-3-*O*-glucoside (Cy-3G) molar extinction coefficient of 25,965 M^−1^·cm^−1^ and a molecular mass of 449.2 g·mol^−1^. Results were expressed as µg cyanidin-3-*O*-glucoside equivalents (CGE) per mL of juice.

### 4.4. Chromatography and Mass Spectrometry

LC-DAD-MS determination of polyphenols was performed on a Waters Acquity H-Class UPLC system (Waters, Milford, MA, USA), including a quaternary solvent manager (QSM), a sample manager with flow through needle system (FTN), a photodiode array detector (PDA) and a single-quadruple mass detector with electrospray ionization source (ACQUITY QDa). Chromatographic analyses were performed on a Waters C18 HSST3 column (100 mm × 2.1 mm i.d., 1.7 μm particle size). PDA detector was set up in the range of 200 to 600 nm. Mass spectrometric detection was set in the negative electrospray ionization mode using nitrogen as nebulizer gas. Analyses were performed in Total Ion Current (TIC) mode in a mass range 50–1000 m/z. Capillary voltage was 0.8 kV, cone voltage 30 V, ion source temperature 120 °C and probe temperature 600 °C.

LC-HR-MS/MS (High Resolution Tandem Mass Spectrometry) measurements were performed on a Dionex Ultimate 3000 UHPLC System, equipped with a quaternary pump, autosampler (100 µL sample loop, partial injection mode, 2 µL injection volume, sample temperature 8 °C), a photodiode array detector (PDA) (Thermo Fisher Scientific, Bremen, Germany). The effluent from PDA detector was connected on-line to an LTQ-Orbitrap Elite mass spectrometer equipped with a high-temperature electrospray ionization (HESI) ion source, controlled by the Excalibur 2.7 software (Thermo Fisher Scientific, Bremen, Germany) and operated in the negative or positive ion mode. The ion spray voltage was set to 4.0 kV, sheath and auxiliary gases on 20 and 5 psi, respectively. The Orbitrap-MS spectra were acquired at the m/z range of 50–2000 with a resolution of 30000. The tandem mass spectra were acquired by collision induced dissociation (CID) in linear ion trap (LIT) at 35% normalized collision energy and isolation width of 2.0 m/z. The fragments were detected at the FT-resolution of 30000. 

For both LC systems, the same chromatographic method was used. Solvent A was 0.1% aqueous HCOOH and solvent B was 0.1% HCOOH in CH_3_CN. The flow rate was 0.5 mL/min and the column temperature was set at 25 °C. Elution was performed isocratically for the first minute with 2% B; from min 1 to min 6 solvent B was linearly increased to 55%; from min 6 to min 10 elution was isocratic with 20% A and 80% B; then, in 0.5 min solvent B was set at 100% and maintained for two minutes. The column was re-equilibrated with 98% A and 2% B before the next injection. An aliquot of the extract dried under vacuum was resuspended in 100 and 10 μL injected through the needle. The identity of the compounds of interest was established by using analytical standards and by combining chromatographic behavior, UV-Vis spectral data, mass spectrometric results and mass fragmentation patterns.

### 4.5. DPPH Free Radical Scavenging Assay

An aliquot of 10 μL of diluted juices in ethanol (0–10 μL/mL final concentration) was added to 1 mL of 30 μM ethanolic solution of stable nitrogen centered free radical 2,2-diphenyl-1-picrylhydrazyl (DPPH•) and the absorbance was monitored spectrophotometrically at 517 nm after 15 min at RT. Radical DPPH• scavenging capacity was estimated from the difference in absorbance with or without antioxidants and expressed as percent DPPH• disappearance as a function of the sample concentration.

### 4.6. Preparation of Aβ_25–35_ Stock Solution

Aβ_25–35_ was dissolved in sterile phosphate buffered saline, pH 7.4 (PBS) at a concentration of 1 mM and incubated in a sonicator bath on ice for 30 min to induce aggregation. After treatment, the solution was incubated overnight at 37 °C and then stored at −20 °C until use. Immediately before treating the cells, the stock solution was diluted to 25 μM final concentration in culture medium.

### 4.7. Cell Culture and Treatment

BV2 (microglial) cells were a kind gift of Prof. Cinzia Fabrizi. Cells were grown in DMEM/F-12 medium containing 10% fetal bovine serum (Gibco BRL Life Technologies Inc., Grand Island, NY, USA) and 2 mM l-glutamine at 37 °C in a humidified atmosphere with 5% CO_2_. Cells were plated at an appropriate density according to each experimental setting and treated with 25 μM aggregated Aβ_25–35_ in the presence or in the absence of 20 μL/mL of extract reconstituted in DMEM/F12. Cells treated only with EtOAc extract were run in parallel. Untreated cells were used as a control.

### 4.8. Cell Viability Assay

Cell viability was determined by using thiazolyl blue tetrazolium bromide (MTT) dye reduction assay. Briefly, cells were seeded in 96-well plates at a density of 3000 cells/well. After treatment, 20 μL of a 5 mg/mL solution of MTT in PBS was added to the culture medium and cells were incubated at 37 °C for 2h. The supernatants were then aspirated off and formazan crystals were dissolved with 100 μL/well of dimethyl sulfoxide. The optical density of each well was determined at 570 nm with a reference at 690 nm using a microplate reader (Appliskan microplate reader, Thermo Scientific, Vantaa, Finland).

### 4.9. qRT-PCR

Total RNA for reverse transcription-polymerase chain reaction (RT-PCR) was extracted using Trizol reagent according to manufacturer’s instructions. Reverse transcription was performed from 1 μg of total RNA using the QuantiTect Reverse Transcription kit as recommended by the manufacturer. Real-time quantitative RT-PCR (qRT-PCR) measurements were performed using an Applied Biosystems 7300 Real-Time PCR System, using ribosomal protein S27a (*RPS27A*) housekeeping gene as normalizing control. The genes of which we evaluated expression levels were heme oxygenase-1 (*HO-1*), interleukin 6 (*IL-6*), interleukin 1-β (*IL-1β*), TNF-α (*TNF-α*), interleukin 4 (*IL-4*), interleukin 10 (*IL-10*) and interleukin 13 (*IL-13*). For this purpose, we chose primers designed with GenScript Real-time PCR Primer Design Software and listed in Table 2.

The amplifications of qRT-PCR were monitored using the SYBR Green fluorescent stain and the presence of a single PCR product was verified by melting curves in all amplifications. The comparative threshold cycle (ΔΔ*C*_t_) method was used to calculate the relative amount of gene expression. The values were normalized with the control and the propagated standard deviations were calculated.

### 4.10. NAD(P)H:Quinone Oxidoreductase 1 (NQO1) Activity

Cells were seeded in 75 cm^2^ flask at a density of 1.5 × 10^6^ cells/flask. After treatment, the cells were washed with PBS and lysed in 250 μL of ice-cold 20 mM Tris-HCl buffer (pH 7.4) containing 250 mM sucrose, 1 mM phenylmethanesulfonyl fluoride and CIP 50×, SigmacOmplete Protease Inhibitor Cocktail by using the Potter-Elvejham homogenizing system. The homogenate was centrifuged at 15,000× *g* for 20 min at 4 °C. NQO1 activity was carried out as previously described [62]. Briefly, the reaction mixture (1 mL) contained 50 mM sodium phosphate buffer (pH 7.4), 0.7 mg/mL bovine serum albumin (BSA), 40 μM 2,6-dichlorophenolindophenol (DCPIP), and 40 μg sample proteins. The reaction was started by adding nicotinamide adenine dinucleotide phosphate reduced form (β-NADPH) at a final concentration of 0.3 mM. The decrease in absorbance, due to the reduction of DCPIP, was monitored at 600 nm during the first 10 s of the kinetics. Unspecific activity was determined by adding 10 μM dicoumarol to the reaction mixture before addition of β-NADPH and subtracted to total activity. NQO1 activity was calculated as nmol of reduced DCPIP per min per mg of total protein by using an extinction coefficient of 21 × 10^−3^ M × cm^−1^. The intracellular NQO1 activity levels were expressed as a percentage compared to control cells.

### 4.11. Nrf2 and NF-κB Immunofluorescence

Cells were seeded on 18 × 18 mm glass coverslips in 6-well plates at a density of 5 × 10^5^ cells/well. After treatment the cells were fixed by incubating with 2% (*w*/*v*) formaldehyde in PBS, washed and permeabilized with 0.1% (*v*/*v*) Triton X-100 in PBS. After washing with PBS, the coverslips were exposed for 30 min to blocking buffer (5% (*w*/*v*) BSA in PBS) and then incubated overnight at 4 °C with the rabbit anti-Nrf2 mAb (1:100 dilution) and with the mouse anti-NF-κB p65 subunit mAb (1:100 dilution) in 1% (*w*/*v*) BSA in PBS. The coverslips were washed with PBS before incubation for 1 h with the secondary antibody Alexa Fluor^®^ 488 goat anti-rabbit IgG (1:500 dilution) and with Texas Red goat anti-mouse IgG (1:100 dilution) in 1% (*w*/*v*) BSA in PBS. Nuclei were stained for 1 min with 1 μg/mL of 4′,6-diamidino-2-phenylindole dihydrochloride (DAPI) in saline solution and the coverslips were mounted with Mowiol. Fluorescence signal was analyzed by recording stained images using an AxioObserver inverted microscope, equipped with the ApoTome System (Carl Zeiss Inc.). Microscopy imaging was performed using the Axiovision software (Zeiss).

To analyze the Nrf2 and NF-κB nuclear translocation by immunostaining assay, ImageJ software was used, and three fields were analyzed for each sample. The average of signal quantification was obtained by the ratio between mean grey value for each nucleus and cytoplasm after subtraction of background signals, according to:

(1) $$\overset{N}{\underset{i=1}{\sum }}\frac{\mathrm{nucleus}-\mathrm{bk}}{\frac{\mathrm{cytoplasm}-\mathrm{bk}}{N}}$$

where the nucleus is the mean grey value for each nucleus measured for each nucleus, cytoplasm is the mean grey value measured for each cytoplasm, bk features the mean of background signals and N = 50. The errors were calculated as propagated standard deviations. 

### 4.12. Fly Stocks and Crosses

Transgenic fly lines that express wild-type human amyloidβ1–42 (w1118; P{UAS-APP.Aβ42.B}m26a #33769) and a pan-glial repo-Gal4 driver (w1118; P{GAL4}repo/TM3, Sb1 #7415) were obtained from Bloomington Drosophila Stock Center (Indiana University, Bloomington, IN). The Ore-R stock used here has been kept in our laboratory for many years.

Briefly, repo-Gal4 virgin females were crossed to Ore-R control flies or Aβ42 flies at 29  °C. The progeny from these crosses were allowed to feed on the diet supplemented with Arabidopsis EtOAc extract (40 μL/mL in standard sugar-yeast medium) for the entire developmental period. For control food, EtOH alone was added.

### 4.13. Climbing Assay

The climbing assay was performed as described in Feiguin and coworkers [63]. Briefly, a group of 10 flies were placed in an empty vial. A horizontal line was drawn 8 cm above the bottom of the vial. The number of flies per group that can climb above the 8 cm mark by 15 seconds after the tap was recorded as the percentage success rate. Ten trials were performed for each group and approximately 100 flies were assayed for each genotype at two time points (at 3–5 days and 10–12 days post-eclosion). All average data are presented as mean ± SEM and compared with 2-tailed unpaired *t*-tests. Statistical tests were performed using Prism (GraphPad Software, Inc.). All behavioral studies were performed at 25 °C.

### 4.14. Statistical Analysis

Experiments were repeated at least in triplicate and all the results are expressed as the mean value ± standard error of the mean (SEM). Statistical comparison between groups was made using unpaired Student’s *t*-test. *p* values < 0.05 were regarded as significant.

## 5. Conclusions

Our data support the evidence that an *Arabidopsis thaliana* extract rich in phenolic compounds exert anti-inflammatory activity through the activation of the Nrf2 pathway. This evidence further underlines the importance of correct nutrition as pointed out by epidemiological studies indicating that a diet rich in fruits, vegetables and spices may afford protection against neurodegeneration. Clinical studies employing anti-inflammatory drugs in AD probably failed to succeed, because the treatment was started when the patients already were in an advanced state of the disease. The nutritional approach may circumvent this problem as supplementation with polyphenols may start even at a young age long before disease onset, allowing both prevention and a delay in disease progression, and supporting drug treatment with standard therapeutic approaches. Taken together, our results open the possibility that the *Arabidopsis thaliana* extract might be useful in the treatment of pathologies that involve chronic inflammation in more complex organisms, such as a mouse, which is one of the most widely used systems to study cellular and molecular bases of human neurodegenerative diseases. Therefore, the anti-inflammatory activity of the polyphenolic extract from *Arabidopsis thaliana* needs to be explored further in additional in vivo studies to provide a possible strategy to modulate an inflammatory response in the CNS.

## Figures and Tables

**Table 1:** Compound identification by combined UPLC-DAD-MS and UHPLC-DAD-HR-MS/MS.

Peak	Compound	Molecular Mass (Calculated)	Molecular Formula	λ Max (nm)	MS^1^ [M-H]^−^ m/z	MS^1^ [M+H]^+^ m/z	MS^2^ [M-H]^−^ m/z	MS^2^ [M+H]^+^ m/z
1 *	Caffeic acid	180.06	C_9_H_8_O_4_	326	179			
2	Quercetin-rhamnoside-hexoside	610.16	C_27_H_30_O_16_	350	609	611	447, 301	449, 303
3	Kaempferol-3-*O*-glucoside-7-*O*-rhamnoside	594.16	C_27_H_30_O_1_5	351	593	595	431, 285	433, 287
4	Quercetin-di-rhamnoside	594.16	C_27_H_30_O_15_	350	593	595	447, 301	449, 303
5	Isorhamnetin-hexoside-rhamnoside	624.17	C_28_H_32_O_16_	345	623	625	315	463, 317
6	Kaempferol-3, 7-di-*O*-rhamnoside	578.17	C_27_H_30_O_14_	351	577	579		433, 287
7 *	Synapic acid	224.06	C_11_H_12_O_5_	327	223			
8–9	di-*O*-sinapoyl-β-glucose (isomers)	592.17	C_28_H_32_O_14_	327	591		367, 349, 223	
10 *	Luteolin	286.05	C_15_H_10_O_6_	350	285	287		

* confirmed by analytical standard injection.

**Table 2:** Primers used in Real-Time PCR experiments.

Gene	Primer Sequence	
	FORWARD	REVERSE
Heme oxygenase-1 (*HO-1*)	AGGAGGTACACATCCAAGCC	TACAAGGAAGCCATCACCAG
Interleukin 6 (*IL-6*)	AAGCTGGAGTCACAGAAGGAG	GGTTTGCCGAGTAGATCTCAA
Interleukin 1-β (*IL-1β*)	TTCGTGAATGAGCAGACAGC	CCATGGTTTCTTGTGACCCT
*TNF-α*	TGGCCTCTCTACCTTGTTGC	GGGAGCAGAGGTTCAGTGAT
Interleukin 4 (*IL-4*)	TGTACCAGGAGCCATATCCA	TTCTTCGTTGCTGTGAGGAC
Interleukin 10 (*IL-10*)	CCCAGAAATCAAGGAGCATT	TCACTCTTCACCTGCTCCAC
Interleukin 13 (*IL-13*)	AGCATGGTATGGAGTGTGGA	TTGCAATTGGAGATGTTGGT
Ribosomal protein S27a (*RPS27A*)	AGAGGCTGATCTTTGCTGGT	ACCAGATGAAGGGTGGACTC

## Supplementary Materials

Supplementary materials can be found at http://www.mdpi.com/1422-0067/20/3/708/s1.

## Abbreviations

Aβ	Amyloid beta peptide
AD	Alzheimer’s disease
DPPH	2,2-Diphenyl-1-picrylhydrazyl
EtOAc	Ethyl acetate
HO-1	Heme Oxygenase 1
NFκB	Nuclear factor kappa-light-chain-enhancer of activated B cells
NQO1	NAD(P)H:Quinone oxidoreductase 1
Nrf2	Nuclear factor E2-related factor 2
PBS	Phosphate buffer saline
ROS	Reactive oxygen species
