# Supplementary material for: Anti-Inflammatory Activity of A Polyphenolic Extract from Arabidopsis thaliana in In Vitro and In Vivo Models of Alzheimer’s Disease
Source: Int J Mol Sci. 2019 Feb 7;20(3):708. doi: 10.3390/ijms20030708 (PMC6387160; doi:10.3390/ijms20030708)
Supplement: Supplementary file 1 [file ijms-20-00708-s001.pdf]

International Journal of
Molecular Sciences

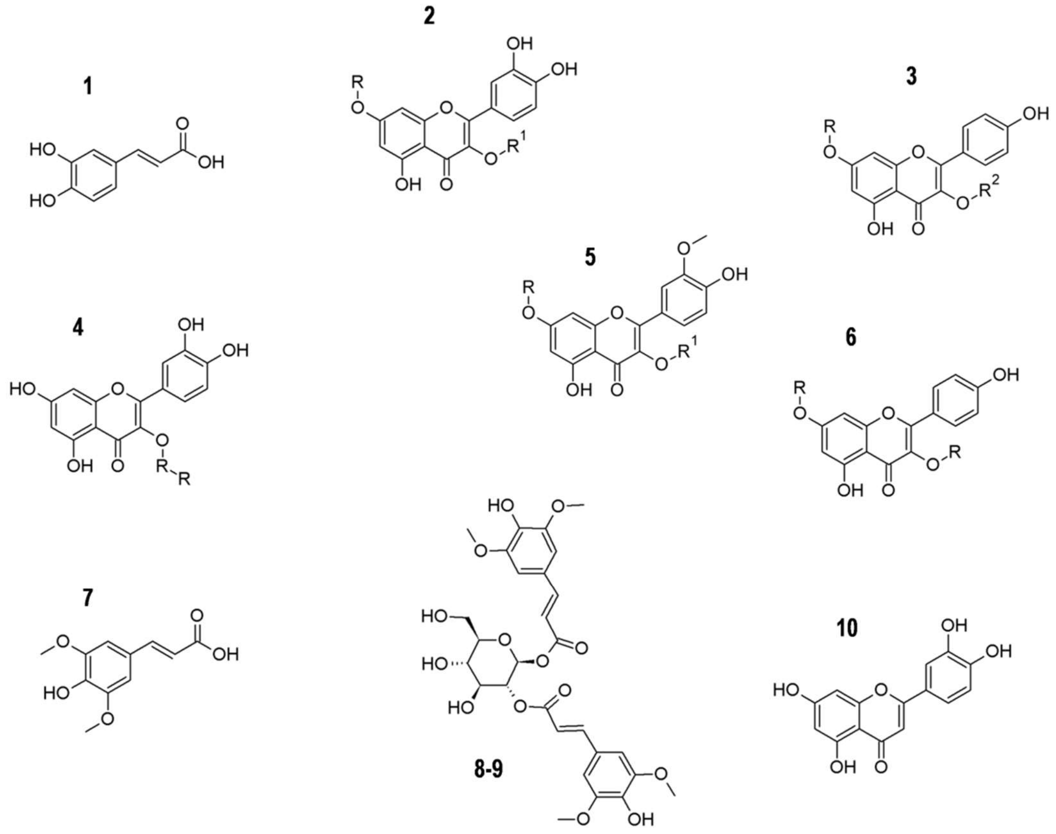

Figure S1. Chemical structures of the most abundant compounds present in *Arabidopsis thaliana* EtOAc extract. R = O-rhamnose; R^1 = O-hexose; R^2 = O-glucose.

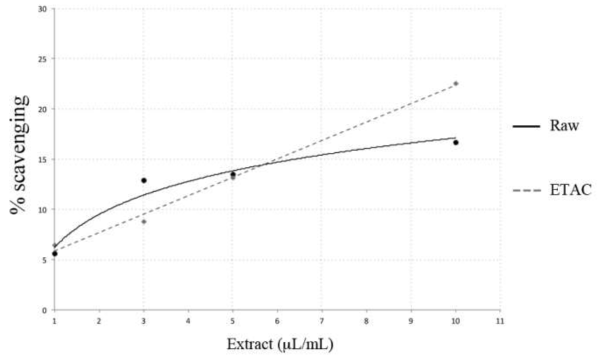

Figure S2. DPPH• scavenging activity of a raw juice and an EtOAc extract from *Arabidopsis thaliana* seedlings. DPPH• scavenging capacity was estimated from the difference in absorbance with or without antioxidants and expressed as percent DPPH• bleaching.
